# Response to: “Quantifying the effect of vaccination on transmission in modelling studies”

**DOI:** 10.1016/j.eclinm.2024.102670

**Published:** 2024-06-06

**Authors:** Sarah M. Bartsch, Kelly J. O'Shea, Ulrich Strych, Maria Elena Bottazzi, Bruce Y. Lee

**Affiliations:** a Public Health Informatics, Computational, and Operations Research (PHICOR), CUNY Graduate School of Public Health and Health Policy, New York City, NY, USA; b Center for Advanced Technology and Communication in Health (CATCH), CUNY Graduate School of Public Health and Health Policy, New York City, NY, USA; c Pandemic Response Institute, New York City, NY, USA; d National School of Tropical Medicine, Department of Pediatrics, and Texas Children's Hospital Center for Vaccine Development, Baylor College of Medicine, Houston, TX, USA; e Department of Molecular Virology and Microbiology, Baylor College of Medicine, Houston, TX, USA

Pan and colleagues^1^ raise several excellent points about the potential for vaccines to reduce transmission in an infected person, pointing out that this would have considerable public health benefits and may be an important outcome measure when developing vaccines. Therefore, we used our computational model of the US population and SARS beta coronavirus transmission^2^ to determine what would happen if a pan-coronavirus vaccine were stockpiled and available at the beginning of an epidemic and provided partial protection against infection, severe disease, and transmission of the circulating coronaviruses. We compared this to a situation in which no vaccines are available and no other interventions are implemented.

Fig. 1 shows how many coronavirus infections are averted when a pan-coronavirus vaccine is the only intervention available and how this varies with vaccine efficacy. With no vaccine, there are 298.4 million infections and 3.6 million deaths,^2^ while vaccinating just 10% of the population (10% vaccine efficacy) resulted in 196.1 million infections and 3.5 million deaths, ultimately saving $6 billion. Our simulations show that a transmission-reducing pan-coronavirus vaccine averts 1.5–2.2 times as many cases and 1.2–1.3 times as many hospitalizations compared to when it only reduces the risk of infection and severe disease (varying with vaccine efficacy [10%–50%] and vaccination coverage [10%–50%]). Such a vaccine is cost-saving in all situations tested, and the additional cost-savings of a transmission-reducing vaccine are 1.2–1.8 times higher.

**Fig. 1:**
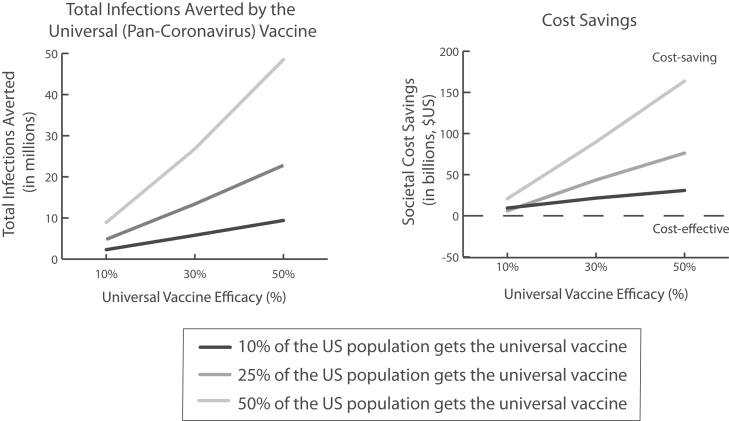
Total infections averted and societal cost-savings achieved by using a universal coronavirus vaccine (costing $60) that reduces the risk of infection, transmission, and severe disease when it is the only intervention available (reproduction number, R_0_, of 2.5) over the course of the epidemic (until there are fewer than 100 cases remaining) and how these outcomes vary with vaccine efficacy (assuming efficacy wanes to 0% within 6 months) and coverage (assuming vaccination coverage is reached 2 months from the epidemic start).

As these additional results show, a vaccine that can also reduce transmission could avert at least 807,000 more cases and save at least $3.4 billion more than if only reducing infection and severe disease, further supporting the development of a pan-coronavirus vaccine. Our model has a few limitations^2^; however, we attempted to be conservative about vaccination's value and did not include longer-term costs due to long COVID-19. While transmission-blocking vaccines are not yet available, efforts are underway to develop mucosal, intranasally administered vaccines that are engineered to reduce viral shedding and thus reduce transmission.^3^ Future vaccine development efforts may want to include transmission blocking as a clinical outcome.

However, a non-transmission-blocking vaccine can still be valuable, especially in situations where no vaccine exists. Our previous work has shown that it is better to vaccinate sooner with a lower-efficacy vaccine than wait for a more efficacious one.^4^ We agree with Pan et al.'s comments that future modeling studies can better inform vaccine development by including the ability to block transmission.

## Contributions

SMB, KJO, and BYL contributed to the model development, data analyses, and interpretation of results. All authors contributed to the writing, editing, and approval of the manuscript. SMB, KO, and BYL accessed and verified the underlying data. All authors agreed to submit the manuscript.

## Declaration of interests

SMB, KJO, and BYL have nothing to disclose. US and MEB report: I am the co-inventor of a protein vaccine technology owned by my employer, Baylor College of Medicine (BCM) that was licensed non-exclusively and with no patent restrictions to several companies committed to advance vaccines for low- and middle-income countries. The co-inventors have no involvement in license negotiations conducted by BCM. Similar to other research universities, a long-standing BCM policy provides its faculty and staff, who make discoveries that result in a commercial license, a share of any royalty income. MEB is a co-director of the Lancet Covid-19 Task Force on Vaccines and Therapeutics.
